# Plasma Membrane Proteomics of Human Breast Cancer Cell Lines Identifies Potential Targets for Breast Cancer Diagnosis and Treatment

**DOI:** 10.1371/journal.pone.0102341

**Published:** 2014-07-16

**Authors:** Yvonne S. Ziegler, James J. Moresco, Patricia G. Tu, John R. Yates, Ann M. Nardulli

**Affiliations:** 1 Department of Molecular and Integrative Physiology, University of Illinois at Urbana-Champaign, Urbana, Illinois, United States of America; 2 Department of Chemical Physiology, The Scripps Research Institute, La Jolla, California, United States of America; Deakin School of Medicine, Australia

## Abstract

The use of broad spectrum chemotherapeutic agents to treat breast cancer results in substantial and debilitating side effects, necessitating the development of targeted therapies to limit tumor proliferation and prevent metastasis. In recent years, the list of approved targeted therapies has expanded, and it includes both monoclonal antibodies and small molecule inhibitors that interfere with key proteins involved in the uncontrolled growth and migration of cancer cells. The targeting of plasma membrane proteins has been most successful to date, and this is reflected in the large representation of these proteins as targets of newer therapies. In view of these facts, experiments were designed to investigate the plasma membrane proteome of a variety of human breast cancer cell lines representing hormone-responsive, ErbB2 over-expressing and triple negative cell types, as well as a benign control. Plasma membranes were isolated by using an aqueous two-phase system, and the resulting proteins were subjected to mass spectrometry analysis. Overall, each of the cell lines expressed some unique proteins, and a number of proteins were expressed in multiple cell lines, but in patterns that did not always follow traditional clinical definitions of breast cancer type. From our data, it can be deduced that most cancer cells possess multiple strategies to promote uncontrolled growth, reflected in aberrant expression of tyrosine kinases, cellular adhesion molecules, and structural proteins. Our data set provides a very rich and complex picture of plasma membrane proteins present on breast cancer cells, and the sorting and categorizing of this data provides interesting insights into the biology, classification, and potential treatment of this prevalent and debilitating disease.

## Introduction

Breast cancer (BC) is the most commonly diagnosed cancer and the second leading cause of cancer-related deaths of women in the United States. It has been estimated that approximately 230,000 women will be diagnosed with BC and 40,000 will die of the disease this year [1].

Although targeted treatments have been developed for tumors that express the estrogen and progesterone receptors or overexpress the ErbB2 protein, these tumors typically develop resistance to currently used treatments. Furthermore, tumors that fail to express any of these proteins, which are classified as triple negative breast cancer (TNBC), have no approved targeted therapeutics. Thus, for both relapsed tumors and TNBCs, the only recourse for treatment is broad spectrum chemotherapy, resulting in debilitating and sometimes persistent side effects.

A recent study using a mathematical model to study cancer treatments and remission indicated that concurrent treatment with two or three different targeted therapies is more likely to induce long-term remission than single or sequential therapies [2]. This concept is illustrated *in vivo* by the phenomenon of kinome reprogramming in TNBC, in which tumor cells ramp up expression of alternate kinases to compensate for the inactivation of a particular receptor tyrosine kinase by targeted treatment [3]. Most importantly, this concept is supported in the clinic by effective treatment of prostate cancer with cabozantinib, which simultaneously targets vascular endothelial growth factor receptor 1 and hepatocyte growth factor receptor [4]. Likewise, simultaneous treatment of melanoma with trametinib, which targets MAP kinase kinase 1, and dabrafenib, which targets the serine/threonine-protein kinase B-raf, has also been successful [5]. Most relevant to BC treatment, dual treatment of ErbB2-positive BC with both the anti-ErbB2 antibody trastuzumab and the tyrosine kinase inhibitor lapatinib resulted in a much higher response rate when compared to administration of either therapy alone [6]. Wider implementation of such dual therapy protocols requires that each tumor be evaluated for diagnostic markers and that a rich library of antibodies and small molecule inhibitors be available to target those markers. Such challenges necessitate the use of novel approaches to define multiple cellular targets, leading to development of pre-clinical paradigms for treatment of refractory BC.

Although targeted therapy is still not widely available, ∼70% of approved targeted drugs and drugs in trials are directed toward plasma membrane (PM) proteins (Table S1). This observation reflects the fact that multiple oncogenic processes are initiated at the PM, including adhesion, proliferation, and migration, and that the PM proteins are more accessible than intracellular targets using the tools and technology currently available.

In order to identify novel PM proteins on BC cells, PMs were prepared from a variety of BC cell lines and subjected to mass spectrometry (MS) analysis. Cell lines were chosen over native tumor tissue in order (i) to provide sufficient material for isolation and analysis of PM proteins, (ii) to avoid problems of tumor heterogeneity, and (iii) to ensure that the proteins we identified were present on BC cells, not endothelial, stromal, adipose, or immune cells. A variety of BC subtypes were examined to maximize the opportunity to observe similarities, differences, and unique expression. Three well-characterized BC cell lines that were originally derived from pleural effusions were chosen for this study including MCF-7 cells (estrogen receptor α and progesterone receptor positive), SK-BR-3 cells (ErbB2 overexpression), and MDA-MB-231 cells (TNBC). MCF-7, SK-BR-3, and MDA-MB-231 cells were chosen because their gene expression profiles are similar to their respective tumor subtypes [7], [8]. Two newer TNBC cell lines derived from primary tumors, DT22 and DT28 cells, which were recently isolated directly from primary tumors, were included to allow investigation of oncogenic processes at the original tumor site [9]. These two cell lines essentially bridge the gap between tumors and cell lines, making them ideal subjects for this type of analysis. MCF-10A cells, which were derived from mammary tissue characterized as benign hyperplasia, served as a normal control.

The current ability of MS to accurately analyze sub-cellular fractions [10] was exploited in this study to maximize identification of candidate target proteins that are accessible at the PM. MudPIT, or Multidimensional Protein Identification Technology, allowed for a rich and sensitive examination of proteins within each of our PM preparations, yielding a PM proteome for each of the cell lines tested. Data were then interrogated to ascertain patterns of expression and explore potential targets for future cancer treatments.

## Materials and Methods

### Cell Lines and Culture

MCF-7, MCF-10A, MDA-MB-231, and SK-BR-3 were originally obtained from ATCC (Manassas, VA). MCF-7 cells were maintained in Eagle's Minimum Essential Medium (MEM) with 5% calf serum (CS), 100 µM non-essential amino acids, and 100 units/ml penicillin/100 µg/ml streptomycin (P/S). MCF-10A cells were maintained in Dulbecco's MEM/F12 with 5% CS, 20 ng/ml epidermal growth factor, 0.5 µg/ml hydrocortisone, 0.1 µg/ml cholera toxin, 10 µg/ml bovine insulin and P/S. MDA-MB-231 and SK-BR-3 cells were maintained in Dulbecco's MEM with 10% fetal bovine serum (FBS) and P/S. Two TNBC cell lines derived from dissociated primary tumors were established from ER-negative primary breast tumors as previously described [9]. Each of these cell lines represents a different molecular subtype within the TNBC category, with DT22 being basal claudin-low and DT28 being basal-epithelial [9]. Both DT cell lines were maintained in Modified Improved MEM with 10% FBS and P/S. All cultures were maintained at 37° in a 5% CO_2_ incubator.

### Plasma membrane isolation

Purified PMs were prepared using differential centrifugation followed by aqueous two-phase partitioning [11], [12]. All steps were carried out at 0–4°C to minimize protease degradation. Briefly, at least 4×10^7^ cells (10 150 mm tissue culture dishes) were washed twice in ice cold PBS, scraped into PBS and pelleted for 5 min at 800×*g*. The cell pellets were resuspended in hypotonic buffer (0.2 mM EDTA, 1 mM NaHCO_3_) with protease inhibitors (Sigma, St. Louis, MO) at the ratio of 10^8^ cells per ml, and the cells were allowed to swell for 10–30 min. Swollen cells were disrupted with 30–50 strokes of a B pestle in a Dounce homogenizer and monitored for cell breakage using trypan blue staining. When cells were more than 90% lysed, nuclei and remaining intact cells were spun out at 800×*g* for 10 min. The supernatant was collected and spun at 100,000×*g* for 1 hour, yielding a cytosolic supernatant and crude total membrane pellet. The pellet was resuspended in 200 mM phosphate buffer pH 7.2 at a ratio of 10^8^ cells per ml, and this mix was subjected to aqueous two-phase partitioning.

The two-phase system was prepared with a 14 g polymer mix of 6.6% dextran T500 (Sigma, St. Louis, MO) and 6.6% w/w polyethylene glycol (Emerald Bio, Bainbridge Island, WA) in 200 mM phosphate buffer, pH 7.2. The membrane suspension was added to the polymers for a final weight of 16 g, and inverted vigorously 40 times to maximize exposure of the membranes to the polymers. The mixture was spun at 1150×*g* and the top phase containing PMs was moved to a clean tube. The bottom phase was re-extracted with fresh top phase and this second top phase was pooled with the first. The pooled top phases were then re-extracted with fresh bottom phase. Finally, the re-extracted pooled top phase was diluted with 5 additional volumes of 1 mM NaHCO_3_ and spun at 100,000×*g* for 1 hr to collect the washed PMs. The membrane pellets were flash frozen on dry ice and stored at −80°C until MS was performed.

### Mass Spectrometry

#### Protein Digestion

All chemicals used for MS were purchased from Thermo Fisher Scientific (Waltham, MA) unless otherwise noted. Deionized water (18.2 MΩ, Barnstead, Dubuque, IA) was used for all preparations.

Pellets from ultracentrifugation were resuspended in 1 ml extraction buffer (635626, Clontech, Mountain View, CA,), followed by precipitation of 100 ug of protein in 23% TCA. Acetone-washed pellets were resuspended in 60 ul digestion buffer (0.1% Rapigest (Waters, Milford, MA) plus 50 mM ammonium bicarbonate) and heated at 60°C for 30 min. Proteins were reduced with 5 mM tris(2-carboxyethyl)phosphine hydrochloride (C4706, Sigma) and alkylated with 10 mM iodoacetamide (Sigma). Proteins were then digested for 18 hr at 37°C in 1 ug trypsin (V5111, Promega, Madison, WI,). Digestion was stopped with addition of 5% formic acid followed by a 30 min incubation at 37°C. Debris was removed by centrifugation, 30 min at 18000×*g*.

#### MudPIT Microcolumn

A MudPIT microcolumn [13], [14] was prepared by first creating a Kasil frit at one end of an un-deactivated 250 µm ID/360 µm OD capillary (Agilent Technologies, Inc., Santa Clara, CA). The Kasil frit was prepared by briefly dipping a 20–30 cm capillary in well-mixed 300 µL Kasil 1624 (PQ Corporation, Malvern, PA) and 100 µL formamide, curing at 100°C for 4 hrs, and cutting the frit to ∼2 mm in length. Strong cation exchange particles (SCX Luna, 5 µm dia., 125 Å pores, Phenomenex, Torrance, CA) were packed in-house from particle slurries in methanol to 2.5 cm. 2 cm reversed phase particles (C18 Aqua, 3 µm dia., 125 Å pores, Phenomenex, Torrance, CA) were then successively packed onto the capillary using the same method as SCX loading.

#### MudPIT analysis

An analytical RPLC column was generated by pulling a 100 µm ID/360 µm OD capillary (Polymicro Technologies, Phoenix, AZ) to 5 µm ID tip. Reversed phase particles (Luna C18, 3 µm dia., 125 Å pores, Phenomenex, Torrance, CA) were packed directly into the pulled column at 800 psi until 15 cm long. The column was further packed, washed, and equilibrated at 100 bar with buffer B (80% acetonitrile 0.1% formic acid) followed by buffer A (5% acetonitrile and 0.1% formic acid). MudPIT and analytical columns were assembled using a zero-dead volume union (Upchurch Scientific, Oak Harbor, WA). LC-MS/MS analysis was performed using an Accela HPLC pump (Thermo) and LTQ XL (Thermo) using an in-house built electrospray stage. Electrospray was performed directly from the analytical column by applying the ESI voltage at a tee (150 µm ID, Upchurch Scientific) directly downstream of a 1∶1000 split flow used to reduce the flow rate to 300 nL/min through the columns. 11-step MudPIT experiments were performed where each step corresponds to 0, 10, 20, 30, 40, 50, 60, 70, 80, 90 and 100% buffer C (500 mM ammonium acetate, 0.1% formic acid, and 5% acetonitrile) being run for 3 min at the beginning of a 110 min gradient. Precursor scanning was performed from 300–2000 m/z. Data-dependent acquisition of MS/MS spectra was performed with the following settings: MS/MS on the 5 most intense ions per precursor scan. Dynamic exclusion settings used were as follows: repeat count, 1; repeat duration, 30 second; exclusion list size, 200; and exclusion duration, 120 seconds.

#### Data Analysis

Protein and peptide identification were done with Integrated Proteomics Pipeline - IP2 (Integrated Proteomics Applications, San Diego, CA. http://www.integratedproteomics.com/) using ProLuCID, DTASelect2. Spectrum raw files were extracted into ms2 files from raw files using RawExtract 1.9.9 (http://fields.scripps.edu/downloads.php) (S12), and the tandem mass spectra were searched against a human protein database (UniprotKB). In order to accurately estimate peptide probabilities and false discovery rates, we used a decoy database containing the reversed sequences of all the proteins appended to the target database (S13). Tandem mass spectra were matched to sequences using the ProLuCID (S14) algorithm with 600 ppm peptide mass tolerance. ProLuCID searches were done on an Intel Xeon cluster running under the Linux operating system. The search space included half and fully tryptic peptide candidates that fell within the mass tolerance window with no miscleavage constraint. Carbamidomethylation (+57.02146 Da) of cysteine was considered as a static modification. DTASelect parameters were —p 2 -y 1 —trypstat —pfp .01 -in.

### RT-PCR

Total RNA was isolated from cultured cells using an RNAqueous kit (Ambion, Life Technologies, Austin, TX) and contaminating DNA was removed using the TURBO DNA-free Kit (Ambion) according to manufacturer's instructions. RNA concentrations were spectrophotometrically measured and cDNA was synthesized from 500 ng total RNA using the iScript cDNA Synthesis Kit (Bio-Rad, Hercules, CA) as described by the manufacturer. The volume of each cDNA reaction was brought to 100 µl and 1 µl of cDNA was combined with iQ SYBR Green Supermix (Bio-Rad, Hercules, CA) and forward and reverse primers for DHRS2 (5′-TGAGACTATTGCCAAGTGGTGAG-3′ and 5′-TCTGAGATGCTTCCTGCTGAATC-3′), KRT17 (5′-CGGAGACAGAGAACCGCTAC-3′ and 5′- ACAATGGTACGCACCTGAC-3′), EPHB4 (5′-GGTCCTGGTGGTCATTGTGG -3′ and 5′-CCGATGAGATACTGTCCGTGTTTG -3′), ICAM1 (5′-CCTATGGCAACGACTCCTTCTC-3′ and 5′-TGTCTCCTGGCTCTGGTTCC-3′), ANPEP (5′-GGCTGCTGTAACGATGAAG-3′ and 5′-TCTGCTGTGCTGTAGGAAG-3′), and KIT (5′-TTGTCCAGGAACTGAGCAGAGATG-3′ and 5′-GCCTTACATTCAACCGTGCCATTG-3′) were added. Quantitative PCR was carried out using a Bio-Rad iQ5 Multicolor Real-Time PCR Detection System and analyzed with the Bio-Rad data analysis module. Standard curves were created and each unknown sample was run in triplicate to obtain relative abundance of each mRNA species.

### Western Blotting

For initial PM isolation using SK-BR-3 cells, samples of input, crude nuclear pellet, cytosol, total membranes, and PMs were taken for downstream analysis, using RIPA buffer (89901, Thermo Scientific) to extract proteins. All purified PM samples used in later analyses were solubilized in 0.5% CHAPS in PBS. The protein concentration of each sample was determined using the bicinchoninic acid (BCA) assay (Thermo Scientific) with bovine serum albumin (BSA) as a standard. 5 µg protein was loaded onto each lane of a denaturing 4–12% gradient gel and fractionated. Proteins were transferred to a nitrocellulose membrane and the blot was probed with an ErbB2- (ab2428, Abcam), prohibitin- (ab28172, Abcam), lamin A/C- (sc20681, Santa Cruz Biotechnologies), calnexin- (sc-11397, Santa Cruz Biotechnologies) or KRT17- (ab51056, Abcam) specific antibody. Western blots were imaged and quantitated with a Licor Odyssey Infrared Imaging System.

### Immunofluorescent Staining

Staining was done essentially as described [15], [16]. Briefly, cells were grown in 8 well chamber slides (Ibidi, Verona, WI), washed once with PBS and fixed in 4% paraformaldehyde for 15 min. at room temperature. The wells were washed 3X with PBS with 100 mM glycine to quench and remove the paraformaldehyde, and permeabilized with 0.1% Triton-X 100 in PBS for 15 minutes. The cells were then incubated in primary blocking buffer (PBS plus 0.05% Tween and 5% normal donkey serum (Jackson Immunoresearch, West Grove, PA)) for at least 15 min. at room temperature. After 3 washes with PBS, the primary antibody, which was diluted in PBS with 0.05% Tween 20, was added to the cells, and samples were incubated overnight at 4°C. Cells were rinsed 3X with PBS with 0.1% Tween 20, secondary antibody was added in PBS with 0.05% Tween 20, and cultures were incubated for 1 hour at room temperature. After 3 washes with PBS with 0.1% Tween 20, nuclei were counterstained with DAPI for 15 minutes at room temperature, samples were washed once with PBS, and fresh PBS was added to each well before imaging.

### Confocal Microscopy

All imaging was performed through the bottom of the chamber slides using a Leica Microsystems TCS SPE high resolution spectral confocal microscope and data was analyzed with Leica Application Suite, Advanced Fluorescence software. Z-stacks were created in 2 micron intervals with 2 passes per xy plane to minimize background.

## Results and Discussion

The overarching goal of these experiments was to use MS to define PM and PM-associated proteins on BC cells most likely to be involved with proliferation and metastasis. Cell lines were chosen over tumor tissue to address the need for adequate starting material for downstream MS and to eliminate the problem of tissue heterogeneity. Also, recent molecular comparisons between hundreds of cell lines and thousands of tumor samples have demonstrated that it is possible to faithfully model tumors using cell lines as long as the choices are genomically informed [17]. In light of this, we selected our cell lines carefully, choosing MCF-7 cells (estrogen receptor α and progesterone receptor positive), SK-BR-3 cells (ErbB2 overexpression), and MDA-MB-231 cells (TNBC) [7], [8]. In addition, two newer TNBC cell lines derived from primary tumors, DT22 and DT28 cells, were included in this study. MCF-10A cells, which were derived from mammary tissue characterized as benign hyperplasia, served as a normal control. MCF-10A cells have been extensively studied for a number of years as a “benign control”, and these cells form polarized, growth-arrested acinar structures when grown in three dimensional culture [18].

**Figure 1:**
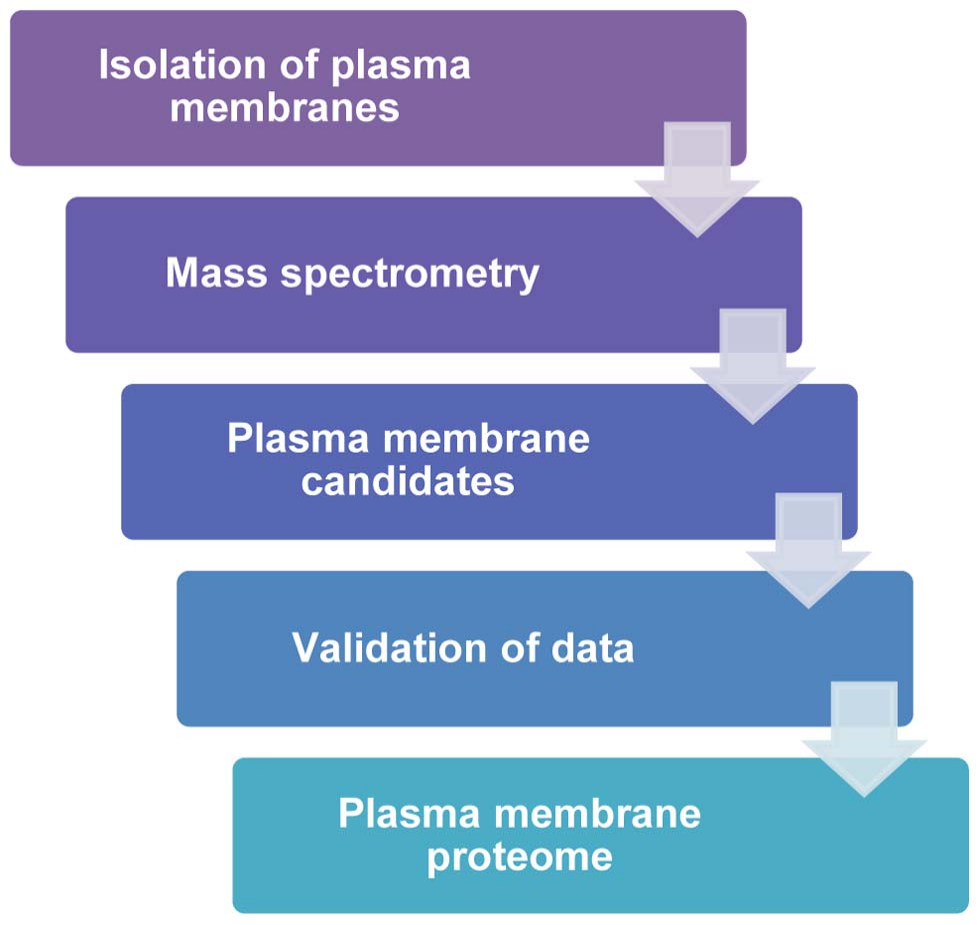
Workflow outlines process of obtaining plasma membrane proteome of multiple BC cell lines. After establishing an effective method for isolating plasma membrane proteins, representatives from each of the currently defined classes of BC were cultured and plasma membranes were isolated and subjected to MS, yielding a comprehensive list of protein identifications having an FDR ≤1%. These data were biologically validated and the data were mined for relevant protein candidates.

**Figure 2:**
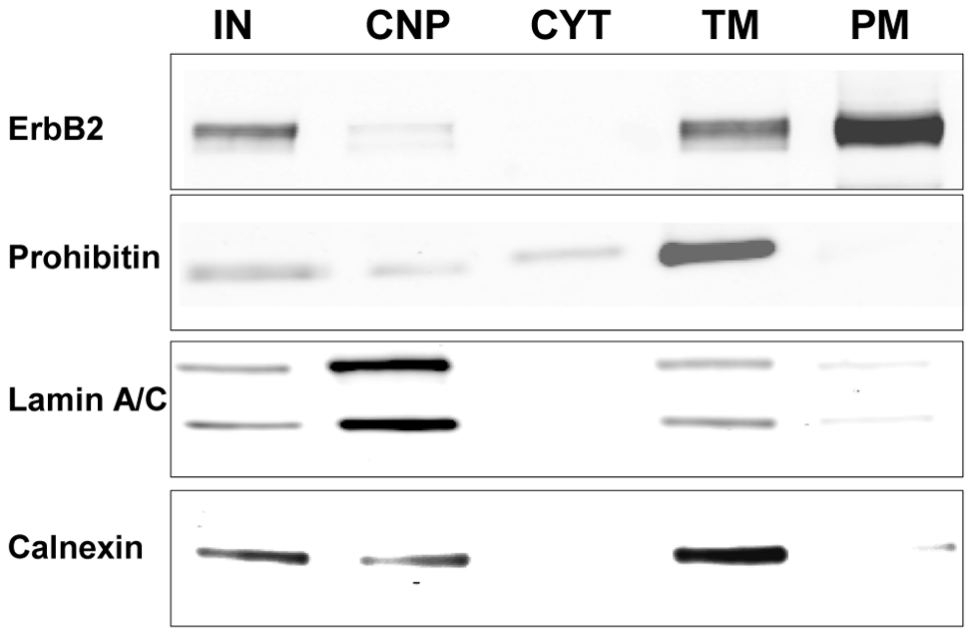
Western blots illustrate the purification of plasma membrane proteins using an aqueous two-phase system. SK-BR-3 plasma membranes were isolated, and the input (IN), crude nuclear pellet (CNP) which also contained unlysed whole cells, cytosol (CYT), total cellular membranes (TM) which included nuclear, mitochondrial, endoplasmic reticular, and plasma membranes, and purified plasma membranes (PM) were subjected to Western blot analysis. ErbB2 is plasma membrane based, prohibitin is anchored in mitochondrial membranes, lamin A/C is found in nuclear membranes, and calnexin is an endoplasmic reticulum protein. All lanes were loaded at 10 µg protein/lane.

Figure 1 outlines the experimental design and workflow used to delineate the PM proteins on these cells. The first step was to find a PM isolation technique that addressed both purity and yield, since both of these parameters are critical for downstream MS analysis. We found that differential centrifugation followed by an aqueous polymer two-phase partitioning method best addressed these requirements [11], [12]. This protocol involved preliminary low and high speed centrifugation steps resulting in the removal of nuclear and cytosolic components, yielding a total membrane fraction that included intracellular membranes as well as PMs. This total membrane fraction was subjected to aqueous two-phase partitioning to separate the PMs from other intracellular membranes. Preliminary experiments were run using SK-BR-3 cells so that the abundant membrane expression of ErbB2 could be followed. As anticipated, Western blot analysis demonstrated that the ErbB2 expressed in SK-BR-3 cells was highly enriched in the PM (Figure 2). Prohibitin, which is present in mitochondrial membranes, was detected in the total membrane preparation, less represented in the input, cytosolic, and crude nuclear fractions, and largely absent in the PM fractions. Also, lamin A/C and calnexin, which are present in nuclear and endoplasmic reticulum membranes, respectively, were essentially excluded from the cytosolic and PM fractions. These results demonstrate the purity of the PMs that were used for downstream MS analysis.

### Comparison of the PM proteomes

MS analysis of the PMs derived from the five BC cell lines and one benign mammary cell line identified a total of 13,650 proteins (FDR ≤1%) from 209,296 spectra (Table 1). DT22 cells appeared to have the most complex membrane, with a total of 7075 proteins, while MCF-10A cells had the fewest with 5660 proteins. Plasma membrane proteomes for each of the cell lines were compared and the number of shared proteins was examined to determine potential relationships (Table 2). Numerically, when comparing the number of proteins in common, the DT22 cells were most similar to the MDA-MB-231 cells, with 4518 shared proteins, and this overt similarity was followed by many more intriguing similarities as the MS data was analyzed. DT28 cells shared the most proteins with SK-BR-3 cells (4048 proteins), and in many ways DT28 cells seemed quite different from the other TNBCs when examining the MS data. MCF-7 cells shared the greatest number of proteins with SK-BR-3 cells, with 4015 proteins in common, but in many ways the MCF-7 PM proteome was quite distinct. The cancer cell lines shared fewer proteins with the benign control MCF-10A cells than with each other.

**Table 1:** Spectral count and protein totals.

Cell line	# spectra	# proteins
**DT22**	40515	7075
**DT28**	29454	5982
**MDA-MB-231**	37815	6483
**SK-BR-3**	33815	6003
**MCF-7**	29542	5704
**MCF-10A**	38155	5660

Each cell line was analyzed for total number of spectra generated and resulting number of proteins identified.

**Table 2:** Intersection of the protein data sets.

Cell line	DT22	DT28	MB-231	SK-BR-3	MCF-7	MCF-10A
**DT22**	**7075**	4023	4518	3852	3539	3382
**DT28**		**5982**	3920	4048	3733	3489
**MB-231**			**6483**	3756	3500	3453
**SK-BR-3**				**6003**	4015	3658
**MCF-7**					**5704**	3493
**MCF-10A**						**5660**

Plasma membrane proteomes for each of the cell lines were compared and the number of shared proteins was examined to determine potential relationships. Bolded numbers reflect the total proteome for the indicated cell line. MDA-MB-231 is abbreviated as MB-231.

**Figure 3:**
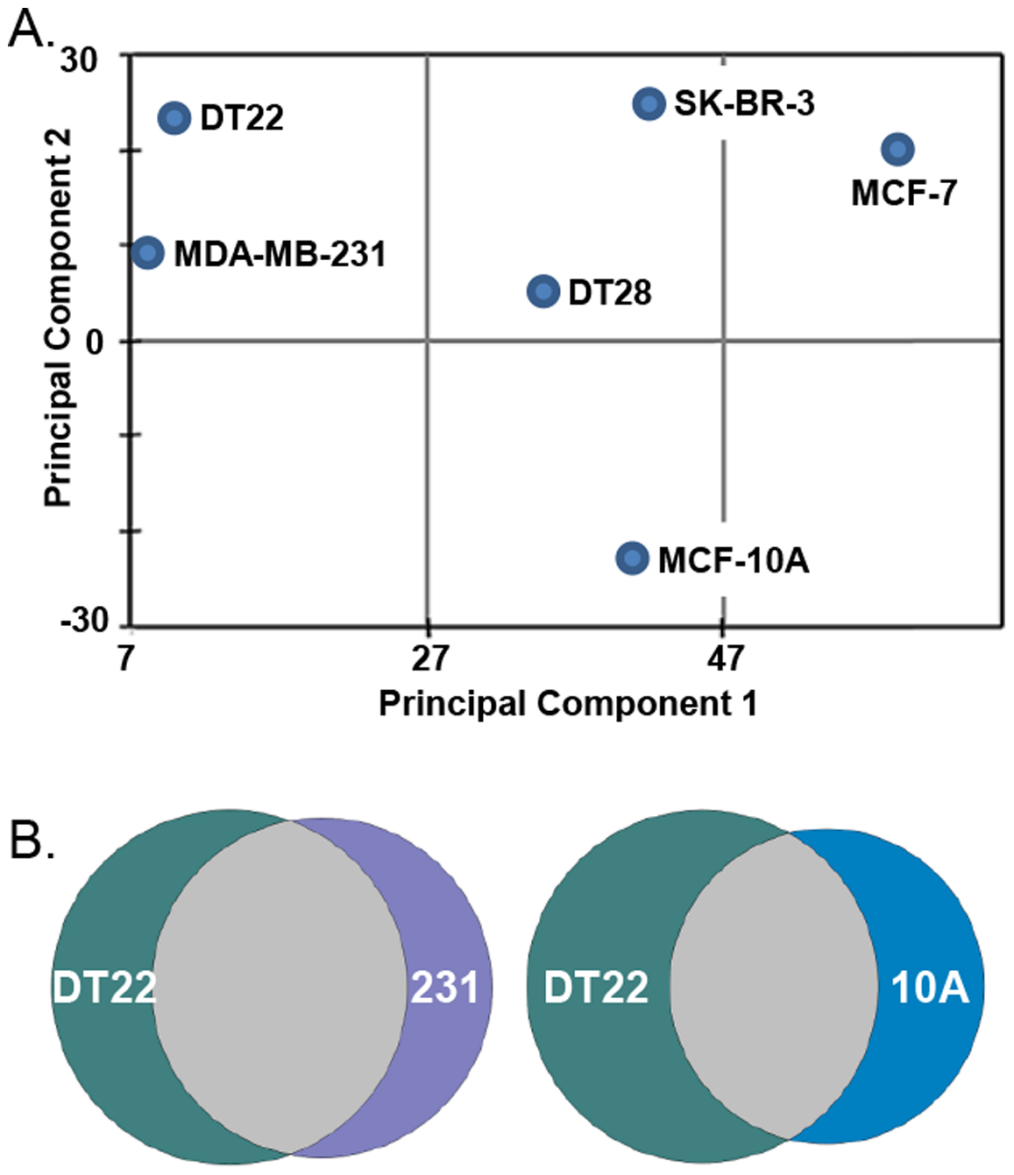
Comparison of PM proteomes shows similarities and differences among the cell lines. A. Relationships between the data sets can be visualized by clustering those that share similar expression profiles. B. Venn diagrams highlight the degree of similarity between DT22 and MDA-MB-231 (231) cells compared to DT22 and MCF-10A (10A) cells.

These relationships can also be visualized using PatternLab, a suite of programs tailored to analysis of shotgun proteomic data [19], [20]. Using this software, relationships among the data sets could be graphically visualized, with a shorter distance between points indicative of a higher degree of similarity (Figure 3A). Again, analysis showed DT22 and MDA-MB-231 PM proteomes to be the most similar whereas the MCF-10A PM proteome had unique characteristics. Venn diagrams also highlight the greater similarity between the DT22 and MDA-MB-231 cells compared to DT22 and MCF-10A cells (Figure 3B).

### Semi-quantitative nature of the MS data

**Figure 4:**
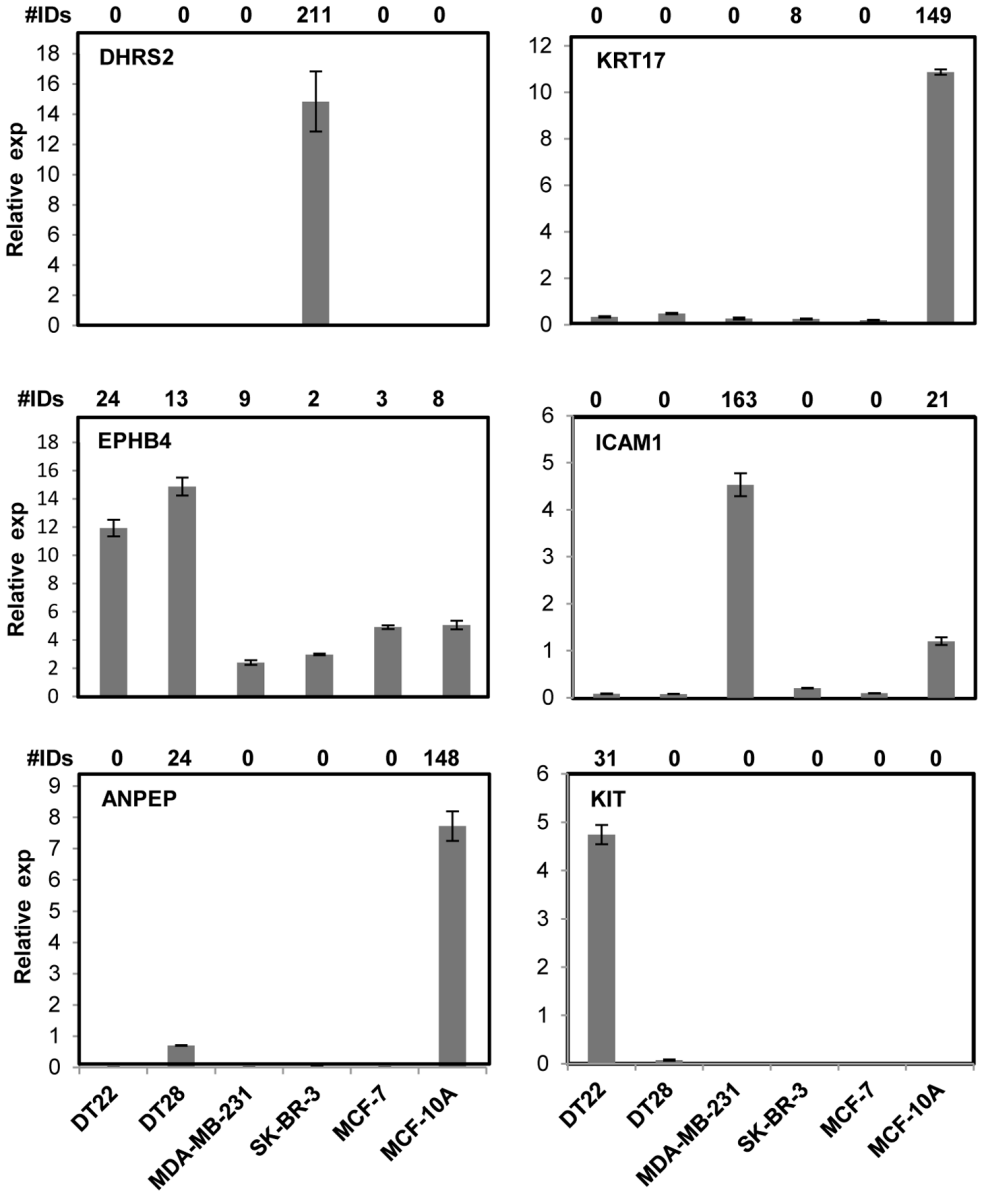
RT-PCR demonstrates the quantitative nature of the MS data. RNA was isolated from each of the cell lines, cDNA was made, and RT-PCR was performed to determine whether the spectral ID numbers were correlated to transcript levels of selected genes. Spectral ID numbers are displayed above each graph and the gene symbol is below those numbers.

**Figure 5:**
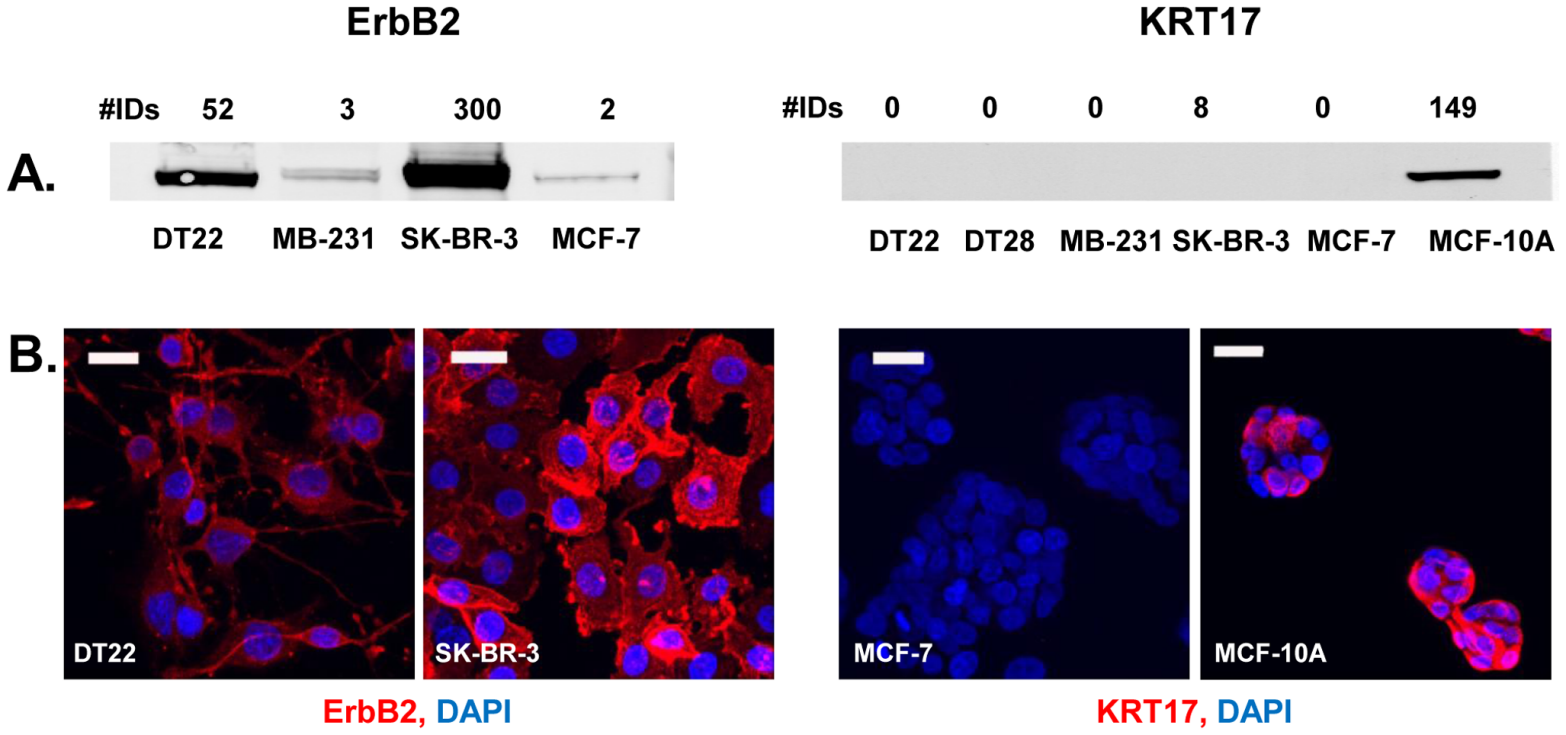
Western blots and immunofluorescence demonstrate that protein levels are accurately reflected in the MS data. A. For Western analysis, PMs were isolated from each of the cell lines, solubilized in detergent buffer, and fractionated on SDS-PAGE gels. Blots were probed with an ErbB2- or KRT17-specific antibody and imaged with the Licor Infrared Imaging System. Spectral ID numbers and cell lines are indicated for each lane on the blot. B. For immunofluorescence, cells were grown in chamber slides, treated with an antibody to ErbB2 or KRT17 (red), and cell nuclei were stained with DAPI (blue). Cells were examined with confocal microscopy. All scale bars  = 25 µ.

Following MS and subsequent data analysis, a lengthy list of PM proteins was generated that required biological validation. We were especially interested in ascertaining the quantitative accuracy of the spectral ID numbers generated, as the techniques used have been shown to yield semi-quantitative data [21]. RT-PCR, Western blot, and immunofluorescent microscopy analyses were used to determine whether the MS data was faithfully reflected in the levels of mRNA and protein present in each of the cell lines. In fact, there was a good correlation between the levels of DHRS2, KRT17, EPHB4, ICAM1, ANPEP, and KIT mRNA levels and the number of spectral IDs (Figure 4). This held true for both uniquely expressed (DHRS2, KRT17, and KIT) and variably expressed (EPHB4, ICAM1, and ANPEP) proteins. An excellent correlation was also observed when comparing spectral ID counts to the presence and intensity of ErbB2 and KRT17 bands on Western blots (Figure 5A). Finally, immunofluorescent microscopy demonstrated good agreement between fluorescent intensity of ErbB2 and KRT17 in intact cells and the spectral ID counts (Panel B).

### Strategies for data analysis

The complexity of our MS data set created multiple opportunities for investigating PM and PM-associated proteins, cell compartments known to be critical for proliferation and metastasis. First, in comparing the BC cell lines to MCF-10A cells, we were able to identify patterns indicative of strategies that BC cells might use to proliferate and metastasize. Second, by utilizing a variety of clinically relevant cell lines we were positioned to discern differences between the various categories of BC. Third, we could compare our BC data to previously described protein expression in other types of cancer, seeking similarities that could both enhance our understanding of cancer and define novel targets for BC treatment that are already being targeted in other types of cancer. Finally, we could define novel targets not yet described in any type of cancer and consider the significance of these targets.

To facilitate examination of the PM data, proteins were grouped and examined by category, and only proteins with higher spectral ID numbers or proteins previously documented to be critical in oncogenesis were included. Categories examined include tyrosine kinases, major histocompatibility class I proteins, cell adhesion molecules, and G protein-coupled receptors (GPCRs).

### Tyrosine kinases

PM-based tyrosine kinases are receptors and transducers for a number of growth factors, cytokines, and hormones that initiate signal transduction cascades which are critical in normal cell biology. However, when de-regulated, these same proteins can fuel several oncogenic processes, thereby promoting proliferation and metastasis. The fact that SK-BR-3 cells had a very high spectral count for the tyrosine kinase ErbB2 was a reassuring indicator of the accuracy of our MS data (Table 3). In addition, some cancer cell lines had significantly higher spectral counts for several other kinases than the benign MCF-10A cells. Surprisingly, the overall expression of these proteins was lowest in MCF-7 cells, even when compared to the MCF-10A cells. The three TNBC cell lines expressed a broad array of tyrosine kinases, suggesting that these cells might exploit multiple signal transduction pathways to fuel growth and metastasis.

**Table 3:** Tyrosine kinases.

Uniprot	*Gene*	DT22	DT28	MB-231	SK-BR-3	MCF-7	MCF-10A	Description
P30530	***AXL***	0	0	58	0	0	0	Tyrosine-protein kinase receptor UFO
P00533	***EGFR***	11	27	89	22	0	11	Epidermal growth factor receptor
P29317	***EPHA2***	0	11	71	0	0	15	Ephrin type-A receptor 2
P04626	***ERBB2***	48	3	3	279	0	0	Receptor tyrosine-protein kinase erbB-2
P21860	***ERBB3***	53	3	0	6	3	0	Receptor tyrosine-protein kinase erbB-3
P08069	***IGF1R***	27	5	5	0	21	5	Insulin-like growth factor 1 receptor
P06213-2	***INSR***	28	0	13	2	2	3	Isoform Short of Insulin receptor
P10721	***KIT***	28	0	0	0	0	0	Mast/stem cell growth factor receptor Kit
Q13308	***PTK7***	32	75	27	0	0	29	Inactive tyrosine-protein kinase 7

Individual tyrosine kinases can interact and function synergistically to promote cancer progression as illustrated by the ability of AXL to network with EGFR and render EGFR-targeted therapy ineffective [22]. Interestingly, MDA-MB-231 cells had the highest number of EGFR spectral IDs and were unique in expressing AXL on their PM. The increased representation of both EGFR and AXL on the PM of MDA-MB-231 cells would predict a failure of these cells to respond to EGFR inhibitors *in vivo,* and clearly illustrates why, in general, drugs targeting tyrosine kinases in TNBCs and other tumors have had disappointing results [3], [22], [23]. Neither DT22 nor DT28 cells had remarkably different EGFR values compared to MCF-10A cells, which is in keeping with a previous report that found approximately 40% of TNBC tumors express EGFR [24].

Another interesting kinase profile was provided by KIT, a cytokine receptor found on the surface of hematopoietic stem cells as well as several types of cancer cells [25]. This kinase was verified to be unique to the DT22 cells by both MS and RT-PCR (Table 3, Figure 4) indicating that KIT could be a potential target in a limited number of breast tumors. Results using IHC in tumor specimens to define the frequency of KIT expression in TNBC are variable, ranging from 30% to 90% of TNBCs examined [26], [27]. The higher representation of the ephrin receptor EPHA2 in MDA-MB-231 cells, ErbB3 in DT22 cells, and PTK7 in DT28 cells indicates that BC cells might exploit multiple signal transduction pathways to fuel growth.

The insulin receptor exists in two isoforms, with isoform B being involved with glucose metabolism and isoform A, in concert with insulin-like growth factor 1 receptor (IGF1R), being involved with cell proliferation [28]. The interplay of isoform A and IGF1R, and the insulin receptor switch in expression from isoform B to isoform A in certain cancer cells, are subjects of great interest in the development of new targeted therapy protocols [29]. Interestingly, DT22 cells had higher spectral IDs for IGF1R and 23 of the 28 spectral IDs for the insulin receptor were unique to isoform A, indicating a proliferative program resulting from the interaction of these two receptors.

When examining the targeted therapies that are currently available for cancer treatment, more than half of them are directed at PM-based tyrosine kinases (Table S1). Interestingly, many of these tyrosine kinase targets were initially described in unrelated types of cancer, including leukemia, non-small cell lung cancer and gastro-intestinal stromal tumors [30]–[32]. This sharing of oncogenic proteins by many diverse cancers lends support to the more recent notion that cancers should be classified by their molecular signatures rather than by their tissue of origin, a concept that could currently expand the number of targeted therapies available for BC treatment.

### Major histocompatibility complex (MHC) class I proteins

MHC class I proteins are present on the PMs of all normal nucleated cells in the body. Their function is to present intracellular peptides on the cell surface for immune surveillance, allowing for detection and destruction of aberrant cells by the immune system. Under ideal circumstances, cancer cells are detected and destroyed because of abnormal peptide displays, but cancer cells can also evolve to down-regulate the abundance of these critical peptide-presenting proteins, lessening the chance for immune system detection and allowing the cancer to progress unchallenged [33]. This appears to be the strategy for DT28, SK-BR-3, and MCF-7 cells, since they have no or very few MHC class I spectral IDs when compared to MCF-10A cells (Table 4). Interestingly, there are some cancers that appear to up-regulate the expression of MHC class I and/or MHC class II molecules [34]–[36]. However, data in this area of study are conflicting, due in part to the lack of correlation between MHC class I RNA transcript levels and MHC class I protein expression on the PM. Also, formalin fixation and paraffin embedding can make MHC class I proteins difficult to detect using IHC staining due to destruction of cell surface epitopes [35]. Thus, MS may be more definitive in providing insights into the expression of these MHC proteins. It is clear from our studies that the DT22 and MDA-MB-231 cells express higher levels of the MHC class I proteins than MCF-10A cells. However, the implications and prognostic value of this expression remain unclear.

**Table 4:** MHC Class I proteins.

Uniprot	*Gene*	DT22	DT28	MB-231	SK-BR-3	MCF-7	MCF-10A	Description		
P05534	***HLA-A***	119	0	57	0	0	13	HLA class I histocompatibility antigen, A-24 alpha chain		
P01892	***HLA-A***	80	0	101	0	0	21	HLA class I histocompatibility antigen, A-2 alpha chain		
P30685	***HLA-B***	202	0	10	0	0	5	HLA class I histocompatibility antigen, B-35 alpha chain		
P18465	***HLA-B***	188	0	25	0	0	0	HLA class I histocompatibility antigen, B-57 alpha chain		
P30508	***HLA-C***	72	0	20	3	0	8	HLA class I histocompatibility antigen, Cw-12 alpha chain		
P61769	***B2M***	104	4	49	3	0	2	Beta-2-microglobulin		

### Cell adhesion molecules

Cell adhesion molecules are proteins on the PM that orchestrate interaction with neighboring cells and the extracellular matrix (ECM) and modulate signal transduction pathways in ways that can profoundly influence cellular behavior. As a result, the most critical characteristics of cancer progression including proliferation, the acquisition of motility, and invasion of the ECM are highly correlated with perturbations in adhesion protein status. Cell adhesion molecules were highly represented in our MS data and included cadherins and catenins, integrins, immunoglobulin superfamily cell adhesion molecules (IgSF-CAMs), two tetraspanins and CD44 (Table 5).

**Table 5:** Cell adhesion proteins.

Uniprot	*Gene*	DT22	DT28	MB-231	SK-BR-3	MCF-7	MCF-10A	Description
**Cadherins and Catenins**								
P12830	***CDH1***	0	6	0	0	5	18	Cadherin-1
P19022	***CDH2***	29	19	0	0	0	2	Cadherin-2
Q9H159	***CDH19***	26	0	0	0	0	0	Cadherin-19
Q9NYQ8	***FAT2***	0	0	0	0	0	22	Protocadherin Fat 2
P35221	***CTNNA1***	172	163	45	21	35	90	Catenin alpha-1
P35222	***CTNNB1***	161	222	58	(2)	22	88	Catenin beta-1
O60716	***CTNND1***	120	118	43	41	23	93	Catenin delta-1
Q02487	***DSC2***	0	4	4	10	0	8	Desmocollin-2
Q14574	***DSC3***	0	4	0	(7)	0	13	Desmocollin-3
Q14126	***DSG2***	0	31	25	9	0	37	Desmoglein-2
P32926	***DSG3***	0	0	0	0	0	24	Desmoglein-3
P15924	***DSP***	0	20	26	24	11	62	Desmoplakin
P14923	***JUP***	24	102	37	38	11	101	Junction plakoglobin
Q99959-2	***PKP2***	0	14	19	3	0	46	Isoform 1 of Plakophilin-2
**Integrins**								
P56199	***ITGA1***	0	0	17	0	0	2	Integrin alpha-1
P17301	***ITGA2***	61	9	125	8	26	107	Integrin alpha-2
P26006	***ITGA3***	7	18	163	3	3	172	Integrin alpha-3
P08648	***ITGA5***	0	25	18	3	5	35	Integrin alpha-5
P23229	***ITGA6***	69	49	53	0	4	118	Integrin alpha-6
P06756	***ITGAV***	93	45	35	29	6	58	Integrin alpha-V
P05556	***ITGB1***	39	28	144	3	15	77	Integrin beta-1
P05106	***ITGB3***	0	0	9	0	0	0	Integrin beta-3
P16144-2	***ITGB4***	0	56	96	0	4	165	Integrin beta, Isoform Beta-4A
P18084	***ITGB5***	3	14	15	18	8	12	Integrin beta-5
P18564	***ITGB6***	0	20	0	6	0	12	Integrin beta-6
**IgSF-CAMs**								
Q13740	***ALCAM***	11	4	96	5	11	44	Activated leukocyte cell adhesion molecule
Q12860	***CNTN1***	59	0	0	0	0	0	Contactin-1
P05362	***ICAM1***	0	0	161	0	0	20	Intercellular adhesion molecule 1
P32004-3	***L1CAM***	115	6	28	9	0	2	Neural cell adhesion molecule L1
P43121	***MCAM***	0	3	140	0	0	2	Cell surface glycoprotein MUC18
P13591	***NCAM1***	65	4	0	0	0	0	Neural cell adhesion molecule 1
**Tetraspanins and CD44**								
P21926	***CD9***	124	21	32	7	12	11	CD9 antigen
P48509	***CD151***	5	7	53	5	0	6	CD151 antigen
P16070	***CD44***	87	13	63	0	0	25	CD44 antigen

Parentheses indicate ambiguity in the protein identification.

Changes in cadherin and catenin expression have previously been observed in breast and numerous other cancers [37]–[40]. These changes include the classic switch from epithelial cadherin (CDH1) to neuronal cadherin (CDH2), and each of the BC cell lines we examined had fewer CDH1 spectral IDs than MCF-10A cells. The increase in CDH2 in both DT22 and DT28 cells is a classic example of BC cells undergoing epithelial to mesenchymal transition (EMT). Unique expression of cadherin 19 by DT22 cells and protocadherin FAT2 by MCF-10A cells was observed, but little is known about these cadherins or their potential roles in cancer. Altered expression of catenins, both lower (MDA-MB-231, SK-BR-3, and MCF-7 cells) and higher (DT22 and DT28 cells) when compared to MCF-10A cells was also observed. Changes in catenin levels have also been correlated with cancer progression. Decreased levels of α-catenin have been associated with poor BC prognosis [37], and MDA-MB-231, SK-BR-3, and MCF-7 cells had fewer spectral IDs for this protein than MCF-10A cells. In contrast, abnormally higher levels of β-catenin are associated with metastasis and poor prognosis [38] due to activation of Wnt signaling and altered expression of multiple genes implicated in cancer progression and metastasis [41]. These actions are thought to be the result of the translocation of β-catenin from the inner PM to the nucleus. MDA-MB-231, SK-BR-3, and MCF-7 cells had a fewer spectral IDs of membrane-associated β-catenin compared to MCF-10A cells, and this fact may reflect this classic situation of a cancer cell losing β-catenin at the PM. The very high number of spectral IDs for both alpha and beta catenin in the DT22 and DT28 PMs is more puzzling.

Desmosomes are cellular structures that orchestrate adhesion to neighboring cells and include the cadherins DSC2, DSC3, DSG2 and DSG3 as well as DSP, JUP, and PKP2. Compared to MCF-10A cells, all of the cancer cells had a lower overall expression of these proteins, illustrating the oncogenic trait of disengagement from surrounding cells. However, the degree of difference was highly variable, ranging from 24 total spectral IDs for DT22 cells to 175 total spectral IDs for DT28 cells. In contrast, MCF-10A cells had 291 total spectral IDs for proteins involved with desmonal attachment.

Integrins are PM proteins involved in the complex interactions between a cell and its ECM including attachment to, remodeling of, and invasion into the matrix [42]. While MS cannot predict the exact α-β dimers that populate the cell surface, it can give an overall impression of the possibilities. Among the cell lines tested here, integrins were differentially expressed both in type and quantity. Interestingly, MDA-MB-231 cells expressed alpha V integrin and were unique in their expression of β-3 integrin, and the αVβ3 dimer is associated with increased tumor aggressiveness [43]. The drug cilengetide targets the αVβ3 dimer and is now in clinical trials for treatment of glioblastoma and other brain tumors, non-small cell lung cancer, squamous cell carcinoma of the head and neck, acute myeloid leukemia, lymphoma, melanoma, Karposi's sarcoma, prostate cancer, and other advanced cancers and solid tumors, including BC (www.clinical trials.gov).

In addition to their overt involvement with adhesion, the immunoglobulin superfamily cell adhesion molecules (IgSF-CAMs) have downstream effects on migration, signal transduction, and gene expression [40]. Several of the IgSF-CAMs stood out as being highly expressed by DT22 and MDA-MB-231 cells (Table 5). Contactin 1 (CNTN1), L1 cell adhesion molecule (L1CAM) and neural cell adhesion molecule 1 (NCAM1) were highly represented in the DT22 cell PM and activated leukocyte cell adhesion molecule (ALCAM), intercellular adhesion molecule (ICAM1), and cell surface glycoprotein MUC18 (MCAM) in the MDA-MB-231 cell PM.

ICAM1, with 161 spectral IDs in the MDA-MB-231 cells, has been successfully targeted with an antibody therapy in a mouse model of multiple myeloma [44]. Furthermore, expression of ICAM1 is highly variable in cancer, being up-regulated in breast, gastric, melanoma, gall bladder, and colon cancers [45], [46] and down-regulated in ovarian [47] and gastric [48] cancers. MCAM is another IgSF-CAM that was uniquely and highly expressed in the MDA-MB-231 cells. Elevated MCAM expression has been correlated with EMT transition, metastasis, and poor prognosis in melanoma, ovarian, prostate, and breast cancers [49], [50]. High expression of L1CAM, which was highly represented on the DT22 PM, has been correlated with poor prognosis in endometrial cancer [51], glioma [52], and non-small cell lung cancer [53]. NCAM1, with significant expression only in DT22 cells, has not been examined in BC, but has been documented as present in 100% of neuroblastomas, rhabdomyosarkomas, gliomas, astrocytomas, and small cell lung cancers, 78% of multiple myelomas, and 53% of acute myeloid leukemias [54]. Some early success using radioimmunoconjugates of anti-NCAM1 to target neuroblastoma and anti-NCAM immunotoxin (lorvotuzumab mertansine) to target small cell lung cancer and multiple myeloma has been reported [54]. CNTN1, which was uniquely expressed by DT22 cells, is associated with loss of E-cadherin expression, metastasis in lung and breast cancer cell lines [55], and poor prognosis in gastic [56] and lung cancer tumors [55]. Overall, the IgSF-CAMs appear to provide a rich field for targeted therapy development. Their over-expression is shared by multiple cancer types including certain BCs.

Tetraspanins CD9 and CD151 have been recently investigated as potential targets in a variety of cancers. CD9 regulates a variety of cellular behaviors including migration, adhesion, and proliferation and interacts with EGFR and integrins in cancer cells [57]. CD9 is up-regulated in ovarian tumors, ovarian cancer cell lines [58], and bone metastases derived from MDA-MB-231 cells [59]. It also mediates chemoresistance in small cell lung cancer [60]. There is currently a preliminary clinical trial investigating the relationship between CD9 expression and leukemia (www.clinicaltrials.gov, Trial ID# NCT01282593). CD151 is expressed in many healthy tissues but has emerged as a potential target because of its association with enhanced cell motility, metastasis, and poor prognosis in cancer. CD151 is over-expressed in lung, colorectal, prostate, pancreatic, breast, and hepatocellular cancers, particularly in advanced disease [61]. Finally, CD44 is another known marker of EMT [62], and both DT22 and MDA-MB-231 cells had significantly higher spectral IDs for this protein, highlighting their potentially aggressive nature.

### GPCRs

GPCRs are integral to signal transduction pathways involving chemokines, hormones and neurotransmitters, and are often differentially regulated and expressed by tumor cells [63]. With more than 2% of the genes encoded by the human genome, GPCRs are the largest family of PM proteins involved in signal transduction. Not surprisingly, they are also receiving increasing attention for their involvement in cancer cell proliferation and metastasis. Although our data were exhaustively interrogated for GPCRs, the only GPCR protein with remarkable ID values was CD97, with aberrantly high expression in DT22 and MDA-MB-231 cells (Table 6). Although this receptor is predominantly expressed by immune system cells, it is up-regulated in certain gastric [64], thyroid [65], prostate [66], colorectal [67], and breast [68] cancers. CD97 has also been correlated with angiogenesis in tumor progression [69].

**Table 6:** GPCRs and G proteins.

Uniprot	*Gene*	DT22	DT28	MB-231	SK-BR-3	MCF-7	MCF-10A	Description
P48960	***CD97***	125	35	96	16	2	6	CD97 antigen
P29992	***GNA11***	82	14	56	10	4	10	Guanine nucleotide-binding protein subunit alpha-11
Q14344	***GNA13***	33	17	27	26	3	9	Guanine nucleotide-binding protein subunit alpha-13
P63096	***GNAI1***	81	20	69	7	5	16	Guanine nucleotide-binding protein G(i) subunit alpha-1
P04899	***GNAI2***	130	32	133	11	7	34	Guanine nucleotide-binding protein G(i) subunit alpha-2
P08754	***GNAI3***	93	26	93	10	13	25	Guanine nucleotide-binding protein G(k) subunit alpha
P09471-2	***GNAO1***	142	18	35	(5)	(2)	(11)	Guanine nucleotide-binding protein G(o) subunit alpha, isoform Alpha-2
P50148	***GNAQ***	66	8	25	6	(2)	10	Guanine nucleotide-binding protein G(q) subunit alpha
P63092	***GNAS***	76	17	30	36	4	15	Guanine nucleotide-binding protein G(s) subunit alpha isoforms short
P62873	***GNB1***	213	52	163	49	12	39	Guanine nucleotide-binding protein G(I)/G(S)/G(T) subunit beta-1
P62879	***GNB2***	240	51	124	41	10	37	Guanine nucleotide-binding protein G(I)/G(S)/G(T) subunit beta-2
P63244	***GNB2L1***	53	42	17	39	33	6	Guanine nucleotide-binding protein subunit beta-2-like 1
Q9HAV0	***GNB4***	113	5	39	(9)	4	7	Guanine nucleotide-binding protein subunit beta-4
Q9UBI6	***GNG12***	7	2	35	2	0	2	Guanine nucleotide-binding protein G(I)/G(S)/G(O) subunit gamma-12

Parentheses indicate ambiguity in the protein identification.

### Proteins affiliated with the PM

Since the protocol utilized to isolate PM proteins was minimally disruptive and used buffers of low ionic strength, a number of proteins associated with the PM were also found in significant numbers in our data set. Therefore, it was not surprising that a large number of cytoskeletal proteins and G proteins appeared in our MS data, since it is known that these proteins physically interact with the PM [70]. However, it is important to keep in mind that the number of spectral IDs representing proteins affiliated with the PM reflect abundance in the cortical region and do not necessarily reflect total cell abundance.

### G proteins

G proteins that directly affiliate with the GPCRs and carry out the next step in signal transduction were well-represented, particularly among the DT22 and MDA-MB-231 cells (Table 6). G proteins with high spectral IDs include GNA11, GNAI1, GNAI2, GNAI3, GNAO1, GNB1 and GNB2. Overall, these proteins have not been well-studied in cancer biology and the correlations with this disease are variable, with some of the G-proteins being up-regulated in cancer, some down-regulated, and one mutated. GNA11 mutation has been implicated in uveal melanoma [71], GNAI1 is down-regulated in hepatocellular carcinoma and has been shown to suppress migration and invasion [72], GNAI2 is down-regulated in ovarian carcinoma [73], high GNAO1 has been correlated with poor prognosis in gastric cancer [74], and GNB1 expression is increased in some BCs [75]. Little is known about GNAI3. In addition, an overall increase in expression of GPCRs and G proteins has been seen in prostate cancer [76]. Overall, we were perplexed by the paucity of differentially expressed GPCRs and the abundance of highly expressed G proteins in our MS data. Paradoxically, the GPCRs and their affiliated proteins are currently targets for 50-60% of all approved drugs [63] but there are few cancer drugs approved or in the pipeline that are in this category [77].

### Cytoskeletal proteins

Proliferation and metastasis require a number of changes in cell morphology and behavior, many of which are driven by dynamic changes in the cytoskeletal protein profile and organization. Many of these changes are initiated at the cell surface as the transformed cell begins to lose polarity and adhesion, take on a more amorphous shape, and assume migratory capabilities [78]. This is reflected in large differences in spectral ID counts found for proteins located in the cortical cytoskeleton which are involved with anchoring the cytoskeleton to the PM (Table 7). The TNBC cells, especially DT22 and MDA-MB-231 cells, had especially high spectral counts in this category and MCF-7 cells had particularly low spectral counts. The three ERM proteins, ezrin, radixin, and moesin, are commonly up regulated in a variety of aggressive cancers and are actively involved with cell signaling and cytoskeletal dynamics at the plasma membrane [79]. These three proteins were more highly expressed in MDA-MB-231, DT22, DT28 and SK-BR-3 cells than in MCF-7 and MCF-10A cells. The three filamins, A, B, and C also had noteworthy expressions. For filamin A, the highest number of spectral IDs was observed in the DT22 cells, but DT28 and MDA-MB-231 cells also expressed high levels of this protein. Filamin A has been correlated with lung tumor growth and angiogenesis [80] as well as metastasis in a variety of cancers [81]. For filamin B, MDA-MB-231 cells had the highest number of spectral IDs, but MCF-10A cells also had significant expression of this protein. Filamin C was uniquely high in MDA-MB-231 cells. The roles of filamins B and C have not been defined in oncogenesis. Alpha-actinin-4 was more highly expressed in all the cancer cell lines compared to MCF-10A, and was particularly high in DT22 and MDA-MB-231 cells. This protein has been implicated in BC tumorigenesis [82].

**Table 7:** Cortical cytoskeleton – proteins anchoring the cytoskeleton to the plasma membrane.

Uniprot	*Gene*	DT22	DT28	MB-231	SKBR3	MCF-7	MCF-10A	Description
P12814	***ACTN1***	123	108	233	60	38	49	Alpha-actinin-1
O43707	***ACTN4***	188	120	312	149	78	58	Alpha-actinin-4
O43491	***EPB41L2***	225	50	48	7	0	8	Band 4.1-like protein 2
P15311	***EZR***	92	87	211	128	28	13	Ezrin
Q9Y4F1	***FARP1***	154	0	23	23	6	35	FERM, RhoGEF and pleckstrin domain-containing protein 1
P21333	***FLNA***	441	162	272	43	50	98	Filamin-A
O75369	***FLNB***	81	51	224	127	39	158	Filamin B
Q14315	***FLNC***	(20)	(11)	84	0	4	5	Filamin-C
P26038	***MSN***	165	114	300	45	8	15	Moesin
P35241	***RDX***	95	67	185	56	8	9	Radixin
Q9Y490	***TLN1***	135	78	171	15	11	2	Talin-1
P46939	***UTRN***	193	22	94	15	0	12	Utrophin

Parentheses indicate ambiguity in the protein identification.

A number of other structural proteins that are affiliated with the cortical cytoskeleton were found in our MS data and many were differentially expressed in the tumor cell lines compared to MCF-10A cells. Categories examined include intermediate filaments, tubulins, actins, and myosins.

### Intermediate Filaments

The aggressiveness of a BC can be predicted by the cytokeratins it expresses [83], [84]. Among these proteins, the most distinct observations included lower overall expression among the TNBC cells, higher overall expression in MCF-7 and SK-BR-3 cells, a much higher expression of KRT17 in the MCF-10A cells, and several keratins that were significantly expressed only in MCF-10A cells (Table 8). The higher expression of KRT17 in MCF-10A cells was confirmed with RT-PCR (Figure 4), Western blot, and immunofluorescence (Figure 5). Vimentin was also examined, since the structural transition from a keratin-rich to a vimentin-rich cytoskeleton has been documented to be most evident in the more aggressive TNBCs [85]. Vimentin spectral IDs were highest in MDA-MB-231 cells, high in DT22 cells, much lower in DT28 and MCF-10A cells, and absent in MCF-7 and SK-BR-3 cells. Using the keratin to vimentin switch as an indicator of aggressiveness, the MDA-MB-231 cells appear to be highly aggressive, as reported [86], and DT22 cells would be predicted to be aggressive as well.

**Table 8:** Structural proteins.

Uniprot	*Gene*	DT22	DT28	MB-231	SK-BR-3	MCF-7	MCF-10A	Description
**Intermediate Filaments**								
P13647	***KRT5***	0	8	0	8	4	50	Keratin, type II cytoskeletal 5
P02538	***KRT6A***	0	4	0	9	4	70	Keratin, type II cytoskeletal 6A
P08729	***KRT7***	0	9	35	20	5	33	Keratin, type II cytoskeletal 7
P05787	***KRT8***	0	3	29	142	103	23	Keratin, type II cytoskeletal 8
P02533	***KRT14***	0	0	0	5	0	13	Keratin, type I cytoskeletal 14
P19012	***KRT15***	0	0	0	5	0	71	Keratin, type I cytoskeletal 15
P08779	***KRT16***	0	0	0	5	0	14	Keratin, type I cytoskeletal 16
Q04695	***KRT17***	0	0	0	6	0	141	Keratin, type I cytoskeletal 17
P05783	***KRT18***	0	12	68	114	164	110	Keratin, type I cytoskeletal 18
P08727	***KRT19***	0	37	31	256	61	0	Keratin, type I cytoskeletal 19
P08670	***VIM***	134	32	321	0	0	20	Vimentin
**Tubulins**								
P68363	***TUBA1B***	70	27	123	31	37	8	Tubulin alpha-1B chain
P07437	***TUBB***	107	62	99	61	39	19	Tubulin beta chain
Q13509	***TUBB3***	36	18	72	13	6	12	Tubulin beta-3 chain
P23258	***TUBG1***	25	8	7	18	10	3	Tubulin gamma-1 chain
Q9UPN3	***MACF1***	55	37	61	4	12	9	Microtubule-actin cross-linking factor 1
P46821	***MAP1B***	2	11	51	0	0	0	Microtubule-associated protein 1B
P27816	***MAP4***	107	43	29	18	17	0	Microtubule-associated protein 4
**Actins**								
P63261	***ACTG1***	795	781	754	544	213	822	Actin, cytoplasmic 2
P68032	***ACTC1***	134	257	106	166	97	216	Actin, alpha cardiac muscle 1
P62736	***ACTA2***	124	143	107	70	34	103	Actin, aortic smooth muscle
**Myosins**								
P35579	***MYH9***	308	121	346	151	13	46	Myosin-9
P35580	***MYH10***	70	13	65	22	13	15	Myosin-10
O43795	***MYO1B***	32	63	55	18	23	133	Unconventional myosin-Ib
O00159	***MYO1C***	132	39	161	94	27	76	Unconventional myosin-Ic
P07951	***TPM2***	11	0	2	0	0	0	Tropomyosin 2 (Beta)
P06753-5	***TPM3***	18	0	0	0	0	0	Tropomyosin 3
P67936	***TPM4***	23	0	6	0	0	0	Tropomyosin alpha-4 chain
O14950	***MYL12B***	35	20	50	3	0	0	Myosin regulatory light chain 12B

### Tubulins

Like the intermediate filaments, the expression of tubulins is frequently altered in cancer cells [87]–[89]. Within the cortical cytoskeleton, DT22 and MDA-MB-231 cells had higher spectral counts for both alpha and beta chains, followed by DT28 and SK-BR-3 cells, then MCF-7 cells. MCF-10A cells were low in tubulin spectral ID numbers (Table 8). The higher spectral ID numbers for TUBB3 in DT22 and MDA-MB-231 cells was of particular interest, since expression of this protein has been associated with chemoresistance and poor prognosis in non-small cell lung cancer [89], [90]. The microtubule-associated proteins MACF1 and MAP1B were both more highly associated with TNBC cells. MACF1 is involved with stabilization of microtubules at the cell surface, facilitation of actin-microtubule interaction at the cell periphery, and positive regulation of Wnt signaling [91]. Little is known about the role of MAP1B in oncogenesis. The microtubule-associated protein MAP4 which promotes microtubule assembly and has been investigated as a potential target in bladder cancer [92], was highly expressed in DT22 cells (107 spectral IDs).

### Actins and Myosins

Actin proteins were highly represented in our data, although MCF-7 cells again appeared to have a significantly lower amount of PM-associated actin than the other BC or benign cells (Table 8).

The altered expression of myosins in a number of cancers [93]–[96] has been correlated with higher motility and metastasis. One myosin, myosin-9 (MYH9), stood out as having very high expression in all the BC cells tested, except for MCF-7 cells, which interestingly expressed a low level of this protein (Table 8). Conversely, unconventional myosin-1b was more highly expressed in MCF-10A cells. MYO1C, which was highly expressed by DT22 (132 spectral IDs) and MDA-MB-231 (161 spectral IDs) cells, is involved with membrane protrusions and actin cytoskeletal recycling [97]. Tropomyosins, which can localize to the cortical cytoskeleton, were more highly expressed only in DT22 cells. TPM2, TPM3, and TPM4 are also up-regulated in a highly aggressive variant of MDA-MB-435 cells [98]. Finally, MYL12B, which was predominantly expressed in the TNBC cells, has been implicated in cytokinesis and cell locomotion [99].

### Oncogenic processes

Although it has been useful to examine the proteins in our MS data set by category, it was also enlightening to interrogate the proteins functionally, looking at processes that are critical for cancer development and metastasis. By doing so, strategies for survival and progression could be investigated and the various types of BC represented here could be compared for potential utilization of those strategies. Proteins with known roles in actin remodeling, invadopodia formation, matrix invasion, lipogenesis, EMT, and PM repair were examined and the BC cell lines were compared for expression of those proteins.

Accelerated turnover of the actin cytoskeleton is known to be involved with metastasis, especially at the leading edge of the cell surface as the cell acquires motility [100]. When examining proteins involved with actin remodeling, DT22 and MDA-MB-231 cells had the highest number of spectral IDs (2978 and 3061 spectral IDs, respectively), followed by DT28 (1677 spectral IDs), SK-BR-3 (1372 spectral IDs), MCF-10A (1185 spectral IDs), and MCF-7 (632 spectral IDs) cells (Table 9, Table S2).

**Table 9:** Oncogenic processes.

PROCESS			DT22	DT28	MB-231	SK-BR-3	MCF-7	MCF-10A
**Actin remodeling**	**# spectral IDs**		2978	1677	3061	1372	632	1185
	**% of total spectral IDs**		7.35	5.69	8.09	4.06	2.14	3.11
**Invadopodia**	**# spectral IDs**		426	264	421	167	73	130
	**% of total spectral IDs**		1.05	0.9	1.11	0.49	0.25	0.34
**Matrix invasion**	**# spectral IDs**		552	265	744	194	57	175
	**% of total spectral IDs**		1.36	0.90	1.97	0.57	0.19	0.46
**Lipogenesis**	**# spectral IDs**		294	198	328	970	308	361
	**% of total spectral IDs**		0.73	0.67	0.87	2.87	1.04	0.95
**EMT**	**# spectral IDs**		1308	716	1727	227	226	532
	**% of total spectral IDs**		3.23	2.43	4.57	0.67	0.76	1.39
**Plasma membrane repair**	**# spectral IDs**		927	613	1605	492	349	625
	**% of total spectral IDs**		2.29	2.08	4.24	1.45	1.18	1.64

Categories relevant to proliferation and metastasis were examined and spectral IDs for proteins in each category were summed for each cell line (Tables S2-S7). This allowed an overall comparison among the cell lines, confirming the more invasive phenotype of the TNBC cells and investigating some specific traits that could potentially be targetable by pharmaceuticals. The % of total spectral IDs was calculated to compensate for differences in the number of spectra obtained for each of the cell lines.

Another way to explore the acquisition of motility is to assess the expression of proteins required for invadopodia formation. Invadopodia are actin-rich specialized sub-cellular structures that incorporate several hallmarks of cancer aggressiveness, including cytoskeletal rearrangements, matrix metalloproteinase secretion aimed at ECM degradation, and tyrosine kinase signaling [101]. The resulting production of small needle-like projections initiates and facilitates the process of ECM invasion. The three TNBC cell lines had higher numbers of spectral IDs related to invadopodia, although DT22 and MDA-MB-231 cells had higher totals than DT28 cells (Table 9, Table S3).

Defining each cancer by its biomarker profile has been gaining traction as more molecular information becomes available about both tumor specimens and representative cell lines. In fact, TNBC has been compared to certain ovarian cancers, and an intriguing study documenting the implantation of metastatic ovarian spheroids into the abdominal mesothelium [96] provided some interesting comparisons to proteins found in our data set. The ovarian cancer cells use physical force generated through cytoskeletal remodeling to literally push the mesothelial cells away from their matrix, allowing the metastasized cells to take their place. The key proteins involved in this invasive process are myosin 9, myosin 10, talin, and integrin α5β1. When spectral IDs for these proteins were tallied for the cell lines in our study, MDA-MB-231 cells had 744 IDs, followed by DT22 (552 spectral IDs), DT28 (265 spectral IDs), SK-BR-3 (194 spectral IDs), MCF-10A (175 spectral IDs), and MCF-7 cells (57 spectral IDs). The relationship between TNBC and ovarian cancer appears to be reflected in our data, particularly by the MDA-MB-231 and DT22 cells (Table 9, Table S4).

Some cancer cells take on a lipogenic phenotype in order to synthesize fatty acids *de novo* for PM expansion and higher energetic requirements [102]. Lipogenesis has been correlated with chemoresistance and an increased protection from both exogenous and endogenous insults [103]. SK-BR-3 cells had a highly elevated number of fatty acid synthase spectral IDs, and it has been reported that this synthase can localize to the PM, even though it is typically defined as a cytoplasmic protein (Human Protein Atlas, http://www.proteinatlas.org). In fact, SK-BR-3 cells stood out from all the other cell lines when examining overall expression of PM and PM-affiliated proteins involved in fatty acid synthesis (Table 9, Table S5). These results indicate that lipogenic capability at the PM may be one of many strategies that cancer cells might employ to promote their survival under adverse conditions.

The EMT is one of the hallmarks of aggressive cancers as the cells lose polarity, dissociate from the ECM and neighboring cells, de-differentiate, and become migratory. Basal BCs, which include the TNBCs, are notorious for expressing this phenotype. The highly metastatic MDA-MB-231 cells stood out as having the most spectral IDs in this category, followed by the cell lines derived from primary TNBC tumors (DT22 and DT28, Table 9, Table S6). SK-BR-3, MCF-7, and MCF-10A cells all had many fewer spectral IDs in this category.

Mechanical stress and immune system attack are both known to cause PM injury [104] and both of these challenges are faced by developing tumors. MDA-MB-231 cells had many more spectral IDs devoted to PM repair than any of the other cell lines (Table 9, Table S7). The potential capability to manage PM integrity may help to explain their aggressive, metastatic phenotype. In fact, knock-down of a critical protein involved in PM repair, myoferlin, causes MDA-MB-231 cells to undergo a mesenchymal to epithelial transition and assume a less invasive phenotype [105], [106].

## Conclusions

Our findings suggest that most cancer cells possess multiple strategies to promote uncontrolled growth, that many of these strategies are initiated at the cell surface, and that the targeting of more than one of these critical proteins simultaneously is currently possible or close to reality. A large library of targeted treatments would be necessary to fully investigate this paradigm as well as implement this as standard of care.

Many potential targets appear to be shared in cancers arising from very different tissues, and, conversely, cancers originating from the same tissue can use very different strategies to promote proliferation and metastasis [107]. These observations confirm the need for personalized medicine when deciding cancer treatment strategies. In more recent years, this idea has come to fruition with treatment of hairy cell leukemia with Vemurafenib, a drug approved for melanoma [108]. Also, certain bone cancers might respond to treatment with erlotinib, an EGFR inhibitor that has been approved for use in lung cancers [109]. Finally, high-grade bladder cancer that over-expresses ErbB2 might be treatable with trastuzumab, a drug targeting ErbB2 in BC [110]. Such fruitful “drug re-purposing” of existing drugs can be the result of carefully designed experiments utilizing high throughput screening of already FDA-approved drugs against a variety of cancer types [111].

In our examination of current targeted therapies in the clinic (clinicaltrials.gov), it was disappointing to discover that the majority of clinical trials to date have not required that a patient's tumor express the protein being targeted. Thus, only a small percentage of the patients who receive a targeted treatment might actually be able to respond to the treatment and many of the trials are far too small to detect such a limited response. Unfortunately, many of the patients enrolled in trials with targeted drugs have advanced disease, which is typically more refractory to treatment. Perhaps most disappointing of all was the fact that the results of the majority of BC trials have never been posted, making it impossible to assess progress on any level. Without the participation of our clinical partners, it will not be possible to enter an era of individualized medicine. Much work remains to be done before targeted therapy with minimal side effects becomes the standard of care and before the outcome of a cancer diagnosis is long-term remission or cure. It is hoped that studies such as this, which reveal potential protein targets on the highly vulnerable cell surface, can move us closer to that goal.

## Supporting Information

Table S1 Targeted Cancer Therapies Currently in Use or Under Investigation. Of the 66 drugs listed, 46 of them target PM proteins (yellow and tan highlight  = 70%) and 34 target membrane-based tyrosine kinases (yellow highlight = 52%). Source: NCI, My Cancer Genome.
(XLSX)

Table S2 Actin remodeling proteins. Parentheses indicate ambiguity in the protein identification.
(XLSX)

Table S3 Proteins involved with creation of invadopodia.
(XLSX)

Table S4 Proteins involved with matrix invasion.
(XLSX)

Table S5 Proteins involved with fatty acid synthesis.
(XLSX)

Table S6 Proteins involved with epithelial to mesenchymal transition.
(XLSX)

Table S7 Proteins involved with plasma membrane repair.
(XLSX)
